# Histoplasmosis or Tuberculosis in HIV-Infected Patients in the Amazon: What Should Be Treated First?

**DOI:** 10.1371/journal.pntd.0003290

**Published:** 2014-12-04

**Authors:** Mathieu Nacher, Antoine Adenis, Emilie Sambourg, Florence Huber, Philippe Abboud, Loïc Epelboin, Emilie Mosnier, Vincent Vantilcke, Julie Dufour, Félix Djossou, Magalie Demar, Pierre Couppié

**Affiliations:** 1 Centre d′Investigation Clinique Antilles Guyane, Inserm CIC1424, Centre Hospitalier de Cayenne, Cayenne, French Guiana, France; 2 Equipe EA3593, Ecosystèmes amazoniens et Pathologie Tropicale, Université de la Guyane, Cayenne, French Guiana, France; 3 Service de Dermatologie Vénérologie, Centre Hospitalier de Cayenne, Cayenne, French Guiana, France; 4 Hôpital de Jour Adultes, Consultations Spécialisées, Centre Hospitalier de Cayenne, Cayenne, French Guiana, France; 5 Unité des Maladies Infectieuses et Tropicales, Centre Hospitalier de Cayenne, Cayenne, French Guiana, France; 6 Département des centres délocalisés de prévention et de soins, Centre Hospitalier de Cayenne, Cayenne, French Guiana, France; 7 Service de Médecine Interne, Centre Hospitalier de l′Ouest Guyanais, Saint Laurent du Maroni, French Guiana, France; 8 Laboratoire Hospitalo-Universitaire de Parasitologie-Mycologie, Centre Hospitalier de Cayenne, Cayenne, French Guiana, France; George Washington University, United States of America

Histoplasmosis and tuberculosis are probably among the most frequent AIDS-defining illnesses in the Amazon region and beyond [1]. Whereas tuberculosis is a well-known disease present in clinical algorithms and in specific public health programs, disseminated histoplasmosis is relatively neglected in South and Central America [2], [3]. Histoplasmosis and tuberculosis are often presented as clinically and paraclinically similar [4]. Recently, we showed that disseminated histoplasmosis, while having some similarities with tuberculosis, had some marked differences with more pulmonary signs and inflammation in tuberculosis whereas histoplasmosis was more likely to be associated with cytopenia, liver enzyme abnormalities, or symptoms from the abdominal sphere [5].

Histoplasmosis and tuberculosis in HIV patients often are disseminated infections with a fatal evolution in the absence of treatment. For both infections, diagnosis is often slow with cultures that may take weeks to isolate the pathogen [6]. Patients with severe disseminated histoplasmosis are at risk of early death within days of their admission, notably if there is treatment delay. For tuberculosis, early mortality in severely immunocompromised patients is also a problem and has led to promote early rather than late initiation of antiretroviral therapy [7].

In practice, once other common opportunistic infections have been excluded, clinicians facing a severely immunocompromised HIV patient will need to conduct investigations and start a presumptive treatment, which often includes antituberculosis drugs but not antifungal drugs. This heuristic of HIV care does not rely on precise epidemiologic data and should be adapted to the local epidemiology.

In French Guiana, HIV is a major public health problem [8]. Histoplasmosisand tuberculosis incidences in HIV-infected patients are high [1], [9], [10]. Therefore, clinicians facing a severely immunocompromised patient often need to consider both alternatives and make a decision.

Since what treatment to start and when to start it may lead to different survival chances in this very common differential diagnosis situation, we aimed to gather additional evidence to guide clinicians.

Longitudinal data from the French Hospital Database on HIV infection (FHDH) in French Guiana between 1996–2008, described in [11], allowed us to collect incidence and mortality rates. The diagnosis of histoplasmosis was performed according to the European Organisation for Research and Treatment of Cancer (EORTC) criteria [12]. The diagnosis of tuberculosis relied on confirmed tuberculosis (culture and identification of *Mycobacterium tuberculosis*). All HIV patients in French Guiana can receive free antiretroviral treatments (including the most recent drugs) regardless of their origin or socioeconomic level.

**Figure 1:**
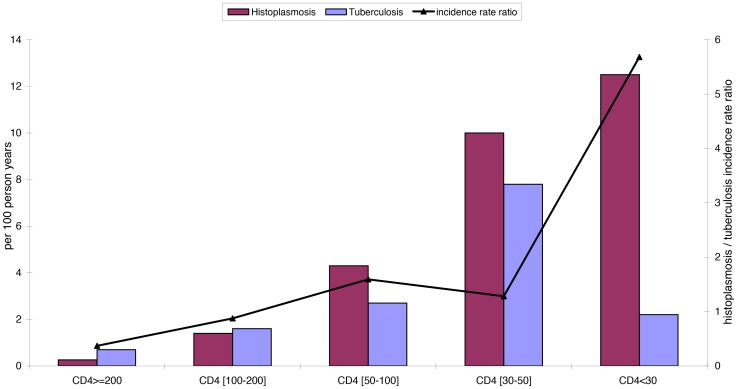
Shows the incidence rate for tuberculosis and histoplasmosis for different CD4 strata.

A total of 2,323 patients were included. This amounted to 40,443 records and 9,608 years at risk. There were 141 first episodes of disseminated histoplasmosis observed and 119 cases of confirmed tuberculosis. Figure 1 shows the incidence rates of first episodes of disseminated histoplasmosis and of tuberculosis for different CD4 strata, and the gradual increase of the incidence rate ratio of histoplasmosis/tuberculosis as immunosuppression increases.

**Figure 2:**
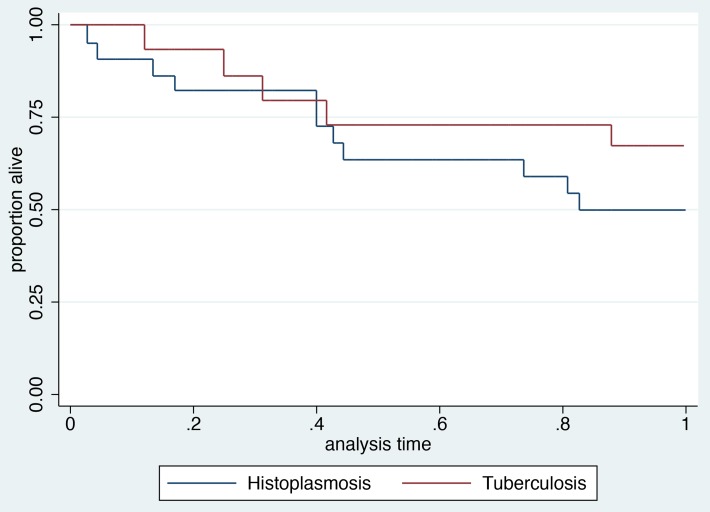
Shows the incidence of death during the first year afer histoplasmosis or tuberculosis among patients with CD4 counts less than 200.

Figure 2 shows the respective Kaplan Meier curves for survival for histoplasmosis and tuberculosis in patients with CD4 counts below 200 cells per mm^3^ within the first 12 months after the opportunistic infection. Histoplasmosis seemed to lead to more deaths than tuberculosis; however, this difference was not statistically significant. For the 141 patients with a first episode of histoplasmosis, there were 13.5% of deaths at one month, 17.5% at three months, and 22.5% at six months after the date of diagnosis of histoplasmosis. Among 119 first episodes of confirmed tuberculosis, 68 were in patients with CD4 counts less than 200 cells per mm^3^. For patients with CD4 counts below 200 cells per mm^3^, there was 10% mortality at one month, 19% at three months, and 31% at six months.

For clinicians, the situation where a severely immunocompromized HIV-infected patient is admitted for a “tuberculosis-like” illness is common and requires prompt identification and treatment of both the opportunistic agent and the underlying immunosuppression. Ideally, treatments should be administered once the opportunistic agent has been identified. However, in the Amazon region, invasive diagnostic procedures are often not performed or available, and laboratory facilities are lacking. Thus, empirical treatment remains an important strategy. Despite the potential adverse events or drug interactions, it is common to simultaneously treat different confirmed or suspected opportunistic infections. However, when possible, it is preferable to target the most likely agent than to give numerous drugs, which makes it difficult to know what leads to improvement or what drug leads to adverse events [13].

In an Amazonian context, among immunosuppressed patients, the incidence of histoplasmosis was higher than that of tuberculosis. Despite comparable overall mortality in terms of proportion of patients with histoplasmosis and tuberculosis dying, the number of histoplasmosis-related deaths was higher. Thus, for HIV patients with CD4 counts below 200 with a tuberculosis-like syndrome (histoplasmosis-like may be a more appropriate heuristic in our epidemiological context), clinicians with poor diagnostic facilities may be better inspired, given the differences in incidence rates, to start with amphotericin B (ideally in its liposomal formulation) than antituberculosis drugs and reevaluate the situation 3–7 days later in view of the treatment response [6], [14]. As shown elsewhere [15], the data also suggests antiretrovirals should be started without delay in order to minimize the duration of the severe immunosuppression that puts the patient at great risk of dying from other opportunistic agents.
